# “Fitness and Fatness” in Children and Adolescents: An Italian Cross-Sectional Study

**DOI:** 10.3390/children8090762

**Published:** 2021-08-31

**Authors:** Matteo Vandoni, Valeria Calcaterra, Vittoria Carnevale Pellino, Annalisa De Silvestri, Luca Marin, Gian Vincenzo Zuccotti, Valeria Tranfaglia, Matteo Giuriato, Roberto Codella, Nicola Lovecchio

**Affiliations:** 1 Laboratory of Adapted Motor Activity (LAMA), Department of Public Health, Experimental Medicine and Forensic Science, University of Pavia, 27100 Pavia, Italy; Matteo.vandoni@unipv.it (M.V.); Vittoria.carnevalepellino@unipv.it (V.C.P.); Luca.marin@unipv.it (L.M.); 2 Pediatric Department, “Vittore Buzzi” Children’s Hospital, 20154 Milan, Italy; valeria.calcaterra@unipv.it (V.C.); gianvincenzo.zuccotti@unimi.it (G.V.Z.); valeria.tranfaglia@unimi.it (V.T.); 3 Pediatric and Adolescent Unit, Department of Internal Medicine, University of Pavia, 27100 Pavia, Italy; 4 Department of Industrial Engineering, University of Rome Tor Vergata, 00133 Rome, Italy; 5 Biometry and Clinical Epidemiology, Scientific Direction, Fondazione IRCCS Policlinico San Matteo, 27100 Pavia, Italy; A.DeSilvestri@smatteo.pv.it; 6 Department of Research, ASOMI College of Sciences, 2080 Marsa, Malta; 7 Department of Biomedical and Clinical Science “L. Sacco”, Università degli Studi di Milano, 20157 Milan, Italy; 8 Unit of Molecular Biology, Department of Health and Natural Sciences, Faculty of Physical Culture, Gdansk University of Physical Education and Sport, 80336 Gdansk, Poland; matteo.giuriato@univr.it; 9 Department of Biomedical Sciences for Health, Università degli Studi di Milano, 20133 Milan, Italy; 10 Department of Endocrinology, Nutrition and Metabolic Diseases, IRCCS MultiMedica, 20138 Milan, Italy; 11 Department of Human and Social Science, University of Bergamo, 24127 Bergamo, Italy; Nicola.lovecchio@unipv.it

**Keywords:** physical fitness test, cardiovascular disease risk factors, children, fatness, fitness

## Abstract

Children with obesity tend to have lower level of physical activity compared to non-obese peers. In fact, sedentary behaviors are prevalent in obese children causing difficulties to perform motor tasks and engaging in sport activities. This, in turn, has direct repercussions on adiposity and related comorbidities. The aim of the study was to investigate several components of fitness and their relationship with the degree of fatness in children. We considered 485 Italian schoolchildren (9.5 ± 1.12 years). BMI and prediction modelling outputs of fat mass were employed as markers of body fatness. Physical fitness (PF) was assessed by the 9-item test battery (explosive power, leg muscle power, arm muscle power, upper body power, coordination, agility, speed and endurance). Differences between groups in the PF tests (*p* < 0.05) were noted. A similar pattern was reflected in both genders. The relationship between anthropometrics’ characteristics and PF tests showed that weight and fat mass had a high level of correlation with different PF tests. Our findings highlight the importance of investigating the degree of fatness in relation with different components of fitness, in children and adolescents. This combination of proxies may cover an unexpectedly helpful screening of the youth population, for both health and performance.

## 1. Introduction

Childhood obesity is a serious public health concern with increasing prevalence in developed countries since, according to the World Health Organization (WHO) report, the global prevalence of overweight in individuals aged 5–19 has risen from 4% in 1975 to 18% in 2016 [1]. Nowadays and worldwide more than 350 million children and adolescents are in a condition of body weight excess. Also, in Italy, 25.2% of children and adolescents from 3 to 17 years old are obese with a higher incidence in the southern regions. Recent studies have demonstrated that obese children tend to be obese adults with long-term medical, social and economic consequences. The excess of weight during childhood contributes to increase the risk of metabolic and cardiovascular diseases such as dyslipidemia, hypertension, and insulin resistance—hallmarks of metabolic syndrome (MetS) [2,3]. This occurs more frequently during adolescence and has been associated with several alterations that limit physical activity (PA) practice with a consequent worsening of the quality of life [2]. Probable mechanisms of obesity-related cardiovascular risk include proinflammatory mediators, metabolic alterations, such as insulin resistance, increased sympathetic nervous system activity, altered vascular function and activation of the renin–angiotensin–aldosterone system [3]. Some studies suggest [4,5] that low levels of physical fitness (PF) (at or below the 25th percentile of battery test outcomes) should be plausibly identified as a “warning sign.” Children with poor PF should be cautiously monitored as this condition may affect their maturation and further physical development [6].

In particular, poor aerobic capacity is associated with a risk of developing MetS or diabetes [7,8] as well as with a risk of future cardiac events [9]. Additionally, low aerobic capacity is a substantial risk factor for a vast range of diseases such as cancer, cardiovascular diseases [10] and diabetes [11], and it is the most powerful predictor of overall mortality equally among healthy people and patients [12,13,14]. Since its benefits were clearly demonstrated, the promotion of regular PA practice and healthy lifestyle became the key element to reduce sedentary behaviors and main cardiovascular disease (CVD) risk factors in children with obesity [5]. Unfortunately, compared to non-obese peers, children with obesity tend to have lower level of PA and prevalent sedentary behaviors. As a result, sport participation is increasingly diminished and even simple motor tasks become difficult. This leads to major implications for the health of the children, including an increased risk of CVD and diabetes [15,16].

One of the most recognized strategies to face this situation is to promote and improve PF among children. PF is defined as the capacity to perform PA developing both physiological and psychological qualities, and is nowadays considered one of the most important health markers, as well as a predictor of morbidity and mortality for CVD [17]. In fact, higher levels of PF, and, in particular, cardiorespiratory fitness, muscular strength and motor fitness in childhood, have been associated with a healthier cardiovascular profile later in life [5]. Evidence suggests that an optimal PF level might attenuate the metabolic impairment associated with excessive fatness [18,19]. Moreover, PF has a positive effect on skeletal and mental health [20,21]. For these reasons, the assessment of PF in children provides relevant information from a clinical and public health standpoint that can be used to maintain and improve children’s health [22]. The most accepted method to assess PF is performing laboratory tests aimed at investigating aerobic domain, strength, power, coordination and agility skills [23,24]. However, these procedures are expensive, time consuming and with a no-ecological approach. To cope with this need, several fitness battery tests were developed in recent years with a high reliability and comparison with direct laboratory tests [25,26]. Fitness and fatness are inversely related as addressed by several studies investigating the main components of PF (mainly, cardiorespiratory fitness and muscular strength). In fact, Ortega et al. [20] reported an inverse relationship between cardiorespiratory fitness & muscular strength and total adiposity in children.

Despite of the validity and reliability of usual physical fitness batteries, most of the included tests were studied in adult populations and then adapted to children [27]. Nevertheless, there is a lack of data of specific tests on children’s motricity such as agility, coordination and strength. Especially in children with poor weight control, these results could be crucial to support an effective exercise prescription. By detecting potential strength and weakness in all domains of PF, exercise specialists can tailor and adapt the exercise proposals to maximize the benefits while rewarding children. In fact, a high level of children’s enjoyment and confident participation in the whole range of physical and sports activities is warranted. Unfortunately, to the best of our knowledge, key components of PF such as lower limb and upper muscular strength, power, coordination, and children weight status are poorly explored and considered altogether.

Therefore, the aim of the present study was to investigate several components of fitness and their relationship with the degree of fatness in children. In addition to body mass index (BMI), as marker of body fatness, we used a novel modelling based on height, weight, age, sex, and ethnicity to predict fat mass in children. It is a simple method relying on simple auxological parameters rather than specific instruments for assessing body composition [28,29].

## 2. Materials and Methods

### 2.1. Study Design and Participants

We conducted a cross-sectional study on a total of 485 Caucasian children (223 girls; 9.5 ± 1.12 years). According to BMI z-score WHO classification [30], children were divided into three groups: underweight (UW; BMI-z score < −2), normalweight (NW; −2 ≤ BMI-z score ≤ 1 and overweight/obese when BMI-z score > 1/2 (OB). Children were recruited from 18 elementary and middle schools in the northern regions of Italy, from the city and hinterland of Milan. All the assessments and tests took place during the curricular physical education (PE) lessons of the Italian academic course and were managed by PE teachers. Inclusion criteria were age between 7 and 12 years, both genders, Italian language proficiency. Exclusion criteria were: any known secondary syndromes with or without obesity, non-comprehension of Italian language, cardiovascular and respiratory chronic diseases, orthopedic injuries, and other absolute, medical impediments to the PA practice given by a registered physician.

After the explanation of all procedures and the involved risks or discomforts, an informed consent was obtained from all parents or legal guardians during the official enrollment while verbal assent was obtained from the children prior to participation. All the participants were free to withdraw from the study at any time. The study protocol was approved by the institutional review board of Regione Lombardia (D.g.r. 9 giugno 2017—n. X/6697) along with the Italian National Olympic Committee (CONI). Protocol study was carried out in accordance with the Declaration of Helsinki of 1975, as revised in 2013 [31]. Each of these public bodies approves projects to be conducted on a national level in accordance with nation regulations and guidelines. The study protocol, including each aspect of the design, was likewise approved by the institutional board of each participating school.

### 2.2. Anthropometric Characteristics

Weight, standing height, and BMI were measured as previously described [16], then fat mass was calculated through a specific formula reported below.

Finally, the fat mass was calculated using the following formula [32]:

Fat Mass = eight − exp(0.3073 × height^2^ − 10.0155 × weight^−1^ + 0.004571 × weight − 0.9180 × ln(age) + 0.6488 × age^0.5^ + 0.04723 × male + 2.8055) (1)

(exp = exponential function, ln = natural logarithmic transformation, male = 1, female = 0)

### 2.3. Physical Fitness Evaluation

PF was assessed by the 9-item test battery, developed by Fjørtoft and colleagues. The test battery [33] was designed to measure explosive power (standing broad jump), leg muscle power (jumping a distance of 7 m on 2 feet, jumping a distance of 7 m on one foot), arm muscle power (throwing a tennis ball with one hand), upper body power (pushing a medicine ball with 2 hands), coordination (climbing wall bars), agility (10 × 5 m shuttle run), speed (running 20 m) and endurance (modified Cooper test; 6 min). In particular, the consistency of the test battery was high (Cronbach alpha value of 0.93) whereas the test-retest reliability was found through Intraclass Correlation Coefficient (ICC) values between 0.54 (Pushing ball medicine) and 0.92 (throwing a tennis ball).

### 2.4. Procedure

All assessments were performed according to the feasibility in the school context and were taken between 8.00 AM and 12.00 AM during the curricular PE lessons in the same environmental conditions (room temperature: 20–24 °C). Test procedures were explained and administered to each child, as already carried out elsewhere [6,34]. Each test was demonstrated from the sport specialist before the children’s attempt. For all tests, three attempts were granted and the best one was considered. The measures were manually collected from the same operator and then inserted into the computer spreadsheet. To guarantee the anonymity, an ID was assigned to all children.

### 2.5. Statistical Analysis

All quantitative data were summarized as mean and standard deviation (SD) and as median and IQR. The Shapiro-Wilk test was used to ascertain the normality of data. Equality of the variances was checked by performing Bartlett’s homogeneity test. Between groups (UW, NW and OB) differences were analyzed by the one-way analysis of variance (ANOVA) with Sheffe’s post-hoc tests. ANOVA was performed also by stratifying groups per gender. To determine possible relationship between markers of body fatness and physical fitness tests, a Pearson correlational analysis was performed. The significance was set at *p*-value less than 0.05. All statistical analysis was performed using Stata software v 16.1 (StataCorp, College Station, TX, USA).

## 3. Results

A total of 471 Caucasian children (215 girls; 9.5 ± 1.12 years) completed the study. Ten children were excluded from the study because parents did not subscribe the informed consent. Four children were excluded from the study because they had orthopedic injuries that did not allow them perform PE lesson. Anthropometrics data of children in the three groups, median values and 25, 75 percentiles are shown in Table 1.

One-way ANOVA highlighted significant differences between groups in the physical fitness tests (*p* < 0.05) except for throwing tennis ball and 5 × 10 m shuttle run tests. All the data are shown in Table 2.

Also, stratifying by gender, significant differences were found for standing broad jump, 7-m two-feet jump (*p* < 0.001), 7-m one-foot jump (*p* < 0.001), 2-hand medicine ball push (*p* < 0.001), wall bars climbing (*p* < 0.001) and 20-m speed run (*p* < 0.001) tests, both in females and in males. Males showed significant differences also in 6-min run test (*p* < 0.001). Females’ data are shown in Table 3, while males’ data are shown in Table 4.

The relationship between anthropometrics’ characteristics and physical fitness tests showed that weight and fat mass had high level of correlation with different physical fitness tests. In particular, body weight showed positive relationship with jumping 7 m on one foot and pushing medicine ball (*p* < 0.05), while it showed an inverse relationship with climbing and 6-min run tests (*p* < 0.05). Also, fat mass showed proportional relationship with jumping 7 m on one foot and throwing tennis ball (*p* < 0.05), while it showed an inverse relationship with climbing and 6-min run tests (*p* < 0.05). All data are shown in Table 5.

## 4. Discussion

In the current study, OB children tend to show a low PF except for upper body power and explosive power (pushing medicine ball and standing broad jump). Conversely, UW children exhibited poor performance as per musculoskeletal power strength and explosive strength. Surprisingly, these data are also supported by the speed performances which resulted to be greater in OB than in UW children. The pattern was reflected in both genders. Furthermore, the correlational analysis appeared to consistently back these data, as positive relationships were confirmed between ponderal indexes (body weight, fat mass, BMI) and explosive performances (ball tennis throws, two feet jumping, medicine ball pushes). On the contrary, children with favorable body shape (low BMI, body weight and stature) succeeded in the agility performances (climbing).

These findings may underscore the determinant role exerted by body mass [35,36,37]. Particularly, this assumption was reinforced by the predictive modelling employed, notwithstanding detailed body composition assessment was not conducted. This design may gain relevance either in clinical settings or in school physical education [17]. To the first end, the practical viability of the predictive method is valuable in a preventive medicine context. As a secondary endpoint, PE teachers should harness whatever child’s physical ability to the maximum level exploitable. This is true when it comes to an obese child that can express strength and power easily, or with a UW child able to better perform in agility.

This educational attitude may build up a virtuous cycle towards higher PF levels, greater enjoyment, self-efficacy, and stronger adherence to active lifestyles, including the participation in a wide variety of sports and physical activities [15,16]. This is an advocated cultural change that is pivotal in current societies as it offers protection against several chronic-degenerative diseases in the adulthood, at no- or low societal costs. Childhood obesity has, in fact, become a major global health problem, since it is associated with various chronic diseases in the short- and long-term, including cardiovascular and metabolic disorders [1,38]. Adiposity promotes cardiovascular risk clustering particularly during pediatric age adolescence [3]. Moreover, poor cardiorespiratory fitness is highly prevalent in children with overweight or obesity and it is also considered as an early predictor of CVD risk factors [39,40]. As reported, a relationship between cardiorespiratory fitness, muscular strength and adiposity has been reported in children [41]. BMI is usually considered a good marker of the cardiometabolic risk [29]. Recently, Licenziati et al. reported [42] that fat mass, assessed by using a new prediction model based on simple anthropometric measures, is associated with subclinical cardiovascular alterations. This model may result useful in preliminarily identifying the cardiovascular risk factors in the early developmental stages of the children, i.e., ahead adulthood, when these conditions become harshly reversible. We reported a moderate correlation between several physical fitness tests and body fatness (BMI and prediction model of fat mass). This confirmed the importance of defining newly parameters for early detection of impaired cardiorespiratory fitness and therefore potential cardiovascular risk. PA and PF levels are inversely related to manifold parameters that indicate health status, including the body fatness. The prevention of overweight and obesity has definitively favorable reflections on PF and overall health.

We are conscious that this study had some limitations. First, we investigated only Caucasian children because they were the majority in the selected schools. Moreover, within this sample selection, confounding factors such as food habits, lifestyle, and ethnicity, that could influence the performance, were excluded. Finally, field-based tests present a lower accuracy of measurement than laboratory setting. However, valid and reliable field-based tests were commonly used to assess the performance in larger samples and showed to be more consistent with environmental conditions. In the future, more in-depth aspects related to gender might be investigated.

## 5. Conclusions

Our findings highlight the importance of investigating the degree of fatness in relation with different components of fitness, in children and adolescents. This simple combination of proxies may cover an unexpectedly helpful screening of the youth population, for both health and performance.

## Figures and Tables

**Table 1:** Anthropometrics’ characteristics of the whole sample.

	UW			NW			OB		
Outcomes	Total (n. 157)	Females (n. 78)	Males (n. 79)	Total (n. 162)	Females (n. 82)	Males (n. 80)	Total (n. 152)	Females (n. 55)	Males (n. 97)
Age (years)	9.72 ± 1.12 (10; 9–11)	9.78 ± 1.10 (10; 9–11)	9.66 ± 1.15 (10; 9–11)	9.47 ± 1.14 (10; 8–10)	9.46 ± 1.16 (10; 8–10)	9.49 ± 1.14 (10; 8.5–10)	9.36 ± 0.90 (9; 9–10)	9.24 ± 0.97 (9; 9–10)	9.42 ± 0.86 (9; 9–10)
Height (m)	1.33 ± 0.08 (1.34; 1.28–1.38)	1.34 ± 0.09 (1.34; 1.29–1.39)	1.33 ± 0.07 (1.34; 1.27–1.38)	1.38 ± 0.07 (1.38; 1.32–1.43)	1.37 ± 0.08 (1.39; 1.32–1.43)	1.38 ± 0.07 (1.37; 1.33–1.43)	1.40 ± 0.08 (1.40; 1.35–1.45)	1.39 ± 0.09 (1.40; 1.33–1.44)	1.40 ± 0.08 (1.40; 1.35–1.45)
Weight (kg)	24.07 ± 3.32 (24; 21.8–26)	24.26 ± 3.78 (24; 21.8–26)	23.88 ± 2.84 (24; 22–26)	33.99 ± 4.59 (34.1; 30.4–36.6)	33.98 ± 4.74 (34.6; 30.4–36)	33.99 ± 4.50 (34; 30.7–36.9)	45.98 ± 7.15 (45; 41–50)	45.40 ± 7.11 (45; 41–50)	46.31 ± 7.28 (45; 41–50)
BMI z-score	−2.59 ± 0.54 (−2.44; −2.78–−2.22)	−2.55 ± 0.47 (−2.4; −2.69–−2.2)	−2.62 ± 0.60 (−2.48; −2.85–−2.2)	−0.08 ± 0.10 (0.07; −0.16–0.02)	−0.09 ± 0.10 (−0.08; −016–−0.01)	−0.07 ± 0.10 (−0.07; −0.16–0.04)	1.43 ± 0.32 (1.35; 1.22–1.57)	1.43 ± 0.31 (1.37; 1.23–1.53)	1.43 ± 0.32 (1.34; 1.22–1.59)
Fat Mass (kg)	4.27 ± 0.62 (4.26; 3.8–4.68)	4.26 ± 0.67 (4.26; 3.8–4.71)	4.28 ± 0.58 (4.26; 3.87–4.65)	9.75 ± 1.57 (9.73; 8.54–10.89)	9.77 ± 1.64 (9.76; 8.54–10.88)	9.72 ± 1.53 (9.54; 8.58–10.67)	17.87 ± 3.78 (17.49; 15.46–19.52)	17.62 ± 3.60 (17.72; 15.14–19.40)	17.99 ± 3.93 (17.39; 15.62–19.71)

n. = numerosity; UW = underweight; NW = normalweight; OB = overweight/obese. All values are shown as mean ± SD (median; 25th–75th percentile).

**Table 2:** Differences between groups in the physical fitness tests.

Groups/Physical Tests	UW Total	NW Total	OB Total
Standing broad jump (cm) Jumping 7 m two feet (sec) Jumping 7 m one foot (sec)	133.49 ± 7.94 *	137.57 ± 7.52 #	139.63 ± 8.17 §
	3.59 ± 0.78 *	3.32 ± 0.58 #	3.66 ± 0.68
	3.31 ± 0.64	3.24 ± 0.61 #	3.57 ± 0.69 §
Throwing tennis ball (m) Pushing medicine ball (m)	13.73 ± 4.58	13.04 ± 4.30	14.19 ± 4.42
	3.65 ± 0.93 *	4.26 ± 1.22	4.33 ± 1.09 §
Climbing (sec) 5 × 10 m shuttle run test (sec) 20 m running (sec) 6 min running (m)	15.39 ± 4.68 *	12.20 ± 4.75 #	14.22 ± 4.70
	22.11 ± 2.90	22.09 ± 3.87	22.15 ± 3.64
	4.55 ± 0.55	4.47 ± 0.58 #	4.75 ± 0.63 §
	963.80 ± 248.02	918.54 ± 164.34	878.92 ± 213.18 §

* Significant difference between UW and NW groups; # significant difference between NW and OB groups; § significant difference between UW and OB groups; sec = seconds; m = meters. All values are shown ad mean ± SD. UW = underweight; NW = normalweight; OB = overweight/obese.

**Table 3:** Differences between groups of female participants in the physical fitness tests.

Groups/Physical Tests	UW Females	NW Females	OB Females
Standing broad jump (cm) Jumping 7 m two feet (sec) Jumping 7 m one foot (sec)	133.91 ± 8.78 *	137.40 ± 7.69 #	138.82 ± 8.59 §
	3.63 ± 0.73 *	3.35 ± 0.56 #	3.68 ± 0.66
	3.32 ± 0.58	3.32 ± 0.58 #	3.59 ± 0.69 §
Throwing tennis ball (m) Pushing medicine ball (m)	15.50 ± 4.24	11.63 ± 3.60	12.31 ± 3.83
	3.49 ± 0.95 *	4.09 ± 0.99	4.01 ± 0.90 §
Climbing (sec) 5 × 10 m shuttle run test (sec) 20 m running (sec) 6 min running (m)	14.51 ± 4.24	12.56 ± 4.77 #	14.84 ± 4.65
	22.63 ± 2.83	22.02 ± 3.74	22.15 ± 3.79
	4.60 ± 0.57	4.45 ± 0.53 #	4.84 ± 0.60 §
	879.03 ± 217.64	906.35 ± 174.01	858.42 ± 243.97

* Significant difference between UW and NW groups; # significant difference between NW and OB groups; § significant difference between UW and OB groups; sec = seconds; m = meters. All values are shown ad mean ± SD. UW = underweight; NW = normalweight; OB = overweight/obese.

**Table 4:** Differences between groups of male participants in the physical fitness tests.

Groups/Physical Tests	UW Males	NW Males	OB Males
Standing broad jump (cm) Jumping 7 m two feet (sec) Jumping 7 m one foot (sec)	133.07 ± 7.04 *	137.75 ± 7.39 #	140.07 ± 7.98 §
	3.56 ± 0.84	3.30 ± 0.59 #	3.65 ± 0.69
	3.29 ± 0.70	3.16 ± 0.63 #	3.56 ± 0.70 §
Throwing tennis ball (m) Pushing medicine ball (m)	14.95 ± 4.61	14.48 ± 4.50	15.28 ± 4.39
	3.81 ± 0.88 *	4.43 ± 1.39	4.52 ± 1.16 §
Climbing (sec) 5 × 10 m shuttle run test (sec) 20 m running (sec) 6 min running (m)	16.18 ± 4.96 *	11.83 ± 4.73 #	13.85 ± 4.72
	21.61 ± 2.89	22.16 ± 4.02	22.16 ± 3.57
	4.51 ± 0.54	4.48 ± 0.62	4.70 ± 0.64
	1046.34 ± 249.22 *	930.88 ± 154.05	890.63 ± 193.90 §

* Significant difference between UW and NW groups; # significant difference between NW and OB groups; § significant difference between UW and OB groups; sec = seconds; m = meters. All values are shown ad mean ± SD. UW = underweight; NW = normalweight; OB = overweight/obese.

**Table 5:** Correlation between anthropometrics characteristics and physical fitness tests.

Correlation between Anthropometrics/Physical Tests	Height (*p*-Value)	Weight (*p*-Value)	BMI z-Score (*p*-Value)	Fat Mass (*p*-Value)
Standing broad jump	0.1984 (0.0045) *	0.1148 (0.1296)	0.0396 (0.5501)	0.1634 (0.0111) *
Jumping 7 m two feet	−0.0541 (0.3890)	0.0291 (0.6431)	0.0460 (0.4640)	0.0429 (0.4949)
Jumping 7 m one foot	−0.0266 (0.6729)	0.1294 (0.0393) *	0.1627 (0.0094) *	0.1728 (0.0508) *
Throwing tennis ball	0.1911 (0.0024) *	0.1042 (0.0995)	0.0379 (0.5499)	0.1532 (0.0151) *
Pushing medicine ball	0.1145 (0.0686)	0.1324 (0.0350) *	0.1633 (0.0098) *	0.1819 (0.0501) *
Climbing	−0.2472 (0.0008) *	−0.2433 (0.0010) *	−0.1942 (0.0088) *	−0.1658 (0.0257) *
5 × 10 m shuttle run test	−0.1043 (0.0959)	0.0030 (0.9624)	0.0550 (0.3808)	0.0030 (0.9624)
20 m running	−0.0548 (0.3829)	0.0737 (0.2400)	0.1163 (0.0631)	0.0737 (0.2400)
6 min running	−0.0491 (0.4427)	−0.2681 (0.0000) *	−0.3217 (0.0000) *	−0.2681 (0.0000) *

sec = seconds; m = meters; * *p* < 0.05.

## Data Availability

The data presented in this study are available on request from the corresponding author. The data are not publicly available due to privacy reasons.
